# Self-Construal Priming Modulates Self-Evaluation under Social Threat

**DOI:** 10.3389/fpsyg.2017.01759

**Published:** 2017-10-13

**Authors:** Tianyang Zhang, Sisi Xi, Yan Jin, Yanhong Wu

**Affiliations:** ^1^School of Public Health and Jiangsu Key Laboratory of Preventive and Translational Medicine for Geriatric Diseases, Medical College, Soochow University, Jiangsu, China; ^2^School of Psychological and Cognitive Sciences, Peking University, Beijing, China; ^3^Beijing Key Laboratory of Behavior and Mental Health, Peking University, Beijing, China; ^4^MOE Key Laboratory of Machine Perception, Peking University, Beijing, China

**Keywords:** self-evaluation, social threat, above-average effect, self-construal priming, social cognition

## Abstract

Previous studies have shown that Westerners evaluate themselves in an especially flattering way when faced with a social-evaluative threat. The current study first investigated whether East Asians also have a similar pattern by recruiting Chinese participants and using social-evaluative threat manipulations in which participants perform self-evaluation tasks while adopting different social-evaluative feedbacks (Experiment 1). Then further examined whether the different response patterns can be modulated by different types of self-construal by using social-evaluative threat manipulations in conjunction with a self-construal priming task (Experiment 2). The results showed that, as opposed to Westerners' pattern, Chinese participants rated themselves as having significantly greater above-average effect only when faced with the nonthreatening feedback but not the social-evaluative threat. More importantly, we found that self-construal modulated the self-evaluation under social-evaluative threat: following independent self-construal priming, participants tended to show a greater above-average effect when faced with a social-evaluative threat. However, this pattern in conjunction with a social threat disappeared after participants received interdependent self-construal priming or neutral priming. These findings suggest that the effects of social-evaluative threat on self-evaluation are not culturally universal and is strongly modulated by self-construal priming.

## Introduction

Human adults evaluate their own personalities and abilities more positively in comparison to how they evaluate other people (Taylor and Brown, 1988; Robins and Beer, 2001; Suls et al., 2002; Chambers and Windschitl, 2004; Sedikides and Gregg, 2008). Previous studies on self-evaluation have employed the social comparison paradigm, which requires participants to assess themselves relative to their peers (Chambers and Windschitl, 2004; Pedregon et al., 2012; Beer et al., 2013). These studies found that the majority of participants reported that they have more positive and less negative qualities and abilities than their average peer. This type of positive illusion of self is currently known as the above-average effect, and it has been demonstrated in a wide range of domains and cultures (Markus and Kitayama, 1991; Yik et al., 1998; Kruger, 1999; Klein and Helweg-Larsen, 2002; Moore and Cain, 2007; Beer and Hughes, 2010).

Interestingly, some recent behavioral studies further found that, when faced with social-evaluative threats, participants tend to evaluate themselves in more flattering ways (Vohs and Heatherton, 2004; vanDellen et al., 2011; Brown, 2012; Beer et al., 2013; Hughes and Beer, 2013). While under social rejection or negative social evaluation conditions, participants evaluated their personalities or abilities more desirably and less undesirably in comparison with nonthreatening conditions. Researchers regard this phenomenon as a fundamental self-protection strategy, which is used to protect the self from the harm pursuant to the social threat (Hughes and Beer, 2013; Hoefler et al., 2015). Several neuroimaging studies have also revealed the neural underpinnings of this fundamental process. They found that self-evaluations made in response to social-evaluative threat increased activation in some regions such as the orbitofrontal cortex (OFC), medial prefrontal cortex (mPFC), and amygdala. Furthermore, these studies further found that the increased desirability under social-evaluative threat was significantly correlated with the medial OFC activity (Flagan and Beer, 2013; Hughes and Beer, 2013). In general, these behavioral and neuroimaging studies have robustly demonstrated that participants respond to social-evaluative threat by emphasizing their own desirability. Only those who suffer from low self-esteem or depression might respond to social threat by evaluating themselves as more undesirable (Vohs and Heatherton, 2004; vanDellen et al., 2011).

Despite these findings, it remains unclear whether the phenomenon of the threatening stimuli increasing desirability is culturally universal or specific to Western participants. Although, the above-average effect has been observed in both populations of Westerners and East Asians (e.g., Yates et al., 1997; Acker and Duck, 2008), several previous studies have found that East Asians have different patterns of self-enhancement from Westerners (Kitayama et al., 1997; Heine et al., 2001; Heine and Hamamura, 2007). Interestingly, previous studies have also found some differences in patterns of self-related processing between East Asians and Westerners when they are faced with social threat or negative stimuli. For example, studies have found that European Americans show a self-face advantage effect during the high-social hierarchical threat condition, while Chinese participants showed a reversal effect (Ma and Han, 2009, 2010; Liew et al., 2011). For this reason, we speculated that East Asians may have different self-evaluation patterns when faced with social-evaluative threat.

In addition, previous studies have found that self-construal differences and self-esteem differences existed among Westerners and East Asians. On one hand, Westerners tend to view the self as an autonomous entity that is separate from others, i.e., independent self-construal; on the other hand, the majority of East Asians tend to view the self as a socially embedded entity with strong interconnectedness with others, i.e., interdependent self-construal (Markus and Kitayama, 1991; Heine, 2001). Although, some researchers regard the phenomenon of responding to social threat by emphasizing one's own desirability as a fundamental self-protection strategy. People with an interdependent self-construal emphasize that augmenting their desirability while under threat is not a wise self-protection strategy because it denies others' negative evaluations about the self by emphasizing one's own desirability and breaking the interconnectedness with others. Moreover, some studies have found that the self-construal priming task can shift self-construals and modulate both Westerners' and East Asians' performances of self-related judgment (e.g., Sui et al., 2009, 2012; Chiao et al., 2010; Kim et al., 2016). Based on these results, we speculated that self-construal differences may modulate self-evaluation responses under social threat. Also, previous studies have found the East-West differences in self-esteem, where East Asians reported lower self-esteem compared to European Americans (Schmitt and Allik, 2005; Chiu and Hong, 2006). In summary, it seems that the potential cultural differences may be caused by the different levels of self-esteem. Therefore, it is necessary to investigate the roles of self-construal and self-esteem in self-evaluation under social-evaluative threat beyond the surface of cultural differences.

In the current study, we aimed to address these questions specifically. The study used social-evaluative threat manipulation and self-construal priming task to assess whether East Asians have different response patterns when faced with social threat and whether these different patterns were caused by different types of self-construal. First, to obtain East Asians' response pattern, we used a typical social-evaluative threat manipulation which has been used in previous Western studies (e.g., Beer et al., 2013; Hughes and Beer, 2013). We manipulated the social-evaluative threat by providing participants with favorable or unfavorable social-evaluative feedback, and then collected their self-evaluation responses (i.e., magnitude of above-average effect). We used the same social-evaluative threat manipulation and self-evaluation procedures for observing the pattern differences between East Asians and Westerners as previous studies. Second, to investigate whether self-construal can modulate self-evaluation under social-evaluative threat, we used a widely-used self-construal priming task (e.g., Sui and Han, 2007; Sui et al., 2012; Wang et al., 2013a,b) in conjunction with different social-evaluative threat manipulations and then asked participants to complete a self-evaluation task. Finally, we also used a standardized self-esteem scale to assess the possible differences in self-esteem (Rosenberg, 1965).

Experiment 1 recruited Chinese participants for investigating the potential cultural differences. We hypothesized that East Asians would engage in different or even opposite patterns of self-evaluation when faced with a social threat. If true, then it would imply that the effect of social threat on self-evaluation is not culturally universal. Experiment 2 then tested whether the differences between Chinese and Westerners were caused by self-construal. We hypothesized that people with different types of self-construal would engage in different patterns of self-evaluation when faced with a social threat. Based on this hypothesis, we predicted that when participants were primed by independent self-construal, the above-average effect should be greater under the threatening manipulation. In contrast, when participants were primed by interdependent self-construal, the above-average effect should have been less when subjected to threatening manipulation; alternatively, there should be no difference between independent self-construal and interdependent self-construal priming.

## Experiment 1

In order to identify whether East Asians have different response patterns when faced with social-evaluative threat, Chinese participants were recruited and we tested the effect of social-evaluative threat on self-evaluation judgments.

### Materials and methods

#### Participants

Thirty-six healthy Chinese participants (19 females and 17 males; *M*_age_ = 21.3, *SD*_age_ = 1.9) participated in Experiment 1. The participants had normal or corrected-to-normal visual acuity. None of them had a history of neurological or psychiatric disorders. The data from an additional six participants were not analyzed because participants expressed suspicion about the threat manipulation at the end of the experiment.

#### Procedures and stimulus materials

A within-subject design was used to manipulate social-evaluative threat while participants made self-evaluation judgments. This experimental paradigm has been used in several previous studies (e.g., Beer et al., 2013; Hughes and Beer, 2013). Two weeks before the target experimental session, participants had their faces photographed and were asked for permission to use their photographs in a cross-university rating study of interpersonal communication. The participants were led to believe that some other college students had evaluated whether they would like to become friends with the participants merely from having observed the photographs. During the social-evaluative threat manipulation, participants received “threatening” or “nonthreatening” feedback. For the “threatening” feedback, participants observed a rating result from a randomly selected set of 10 raters (5 male, 5 female), with most of the raters determining that they would not like to become friends with the participant (6, 7, or 8 of the 10 raters). Similarly, for the “nonthreatening” feedback, participants also observed a rating result from 10 raters, but all of the raters determined that they would like to become friends with the participant. While there were no actual raters, all photographs were selected from a set of 60 photographs of college-aged students collected in the pilot experiment. After receiving social-evaluative threat manipulation in each condition, participants were then instructed to evaluate how they compared with their average peer on personality trait words using a 5-point scale (+2 = much more than the average peer; +1 = slightly more than the average peer; 0 = no difference from the average peer; −1 = slightly less than the average peer; −2 = much less than the average peer). To ensure that the “average peer” was essentially comparable, we recruited college students and instructed them to evaluate their personality traits compared to the average college student of their age and gender.

Across the experiment, there were 3 threatening and 3 nonthreatening pieces of feedback, which were randomly presented. After each feedback session, there was a block of 10 personality traits needing to be evaluated (a total of 60 personality trait words randomly presented and counterbalance across social-evaluative threat manipulation: 30 for threatening, 30 for nonthreatening). All trait words were undesirable words (e.g., bossy, messy, pessimistic, timid, stingy) selected from previous studies (Zhu et al., 2007; Hughes and Beer, 2011). The experiment was conducted in a dimly lit room, in which the participants sat approximately 60 cm away from a monitor (1,440 × 900 pixel resolution). The participants' responses were recorded using a mouse. At the end of the experiment, the Rosenberg Self-esteem Scale (RSES; Rosenberg, 1965), a 10-item instrument, was used to assess self-esteem. The scale has been adopted in a Chinese population and demonstrated good reliability (Martin et al., 2006). After the experiment, participants received a debriefing interview procedure used in a previous study (Hughes and Beer, 2013), which ensured that the participants really believed in our social-evaluative threat manipulation and had no suspicions about the experiment.

### Results

The descriptive statistics of the measures for self-esteem were summarized in Table 1. Consistent with the previous Western studies, all participants were within the normal range of self-esteem (Rosenberg, 1965). More importantly, the magnitude of the above-average effect (the mean reverse-scored value of personality trait words evaluation, which indicates increased desirability) in the self-evaluation task was calculated for two experimental conditions (threatening or nonthreatening feedback) and was submitted to a paired samples *t*-test. The results showed that, for the threatening feedback condition, the magnitude of the above-average effect was significantly less than nonthreatening feedback condition, *t*_(35)_ = −2.62, *p* < 0.05, Cohen's *d* = 0.44 (see Table 1). These findings indicate that, as opposed to Western participants' pattern, the Chinese participants rated themselves as having significantly less undesirable personality traits only when they were faced with the nonthreatening feedback.

**Table 1:** Means and Standard deviations for experiment 1–2.

	**SES**	**Threat**	**No threat**
**Measure**	**Mean (SD)**	**Mean (SD)**	**Mean (SD)**
No priming (Chinese)	32.00 (3.32)	0.38 (0.52)	0.51 (0.49)
Independent priming	33.11 (4.11)	0.56 (0.36)	0.41 (0.33)
Interdependent priming	34.37 (2.40)	0.43 (0.42)	0.60 (0.39)
Neutral priming	32.74 (3.70)	0.42 (0.53)	0.53 (0.49)

*Mean, the magnitude of the above-average effect (the mean reverse-scored value of personality trait words evaluation, which indicates increased desirability); SD, standard deviation*.

## Experiment 2

In order to further identify whether the differences between Chinese and Westerners were caused by different types of self-construal, Experiment 2 was built on Experiment 1 by testing the crossed effects of social threat and self-construal on self-evaluation. The experiment drew on experimental procedures used in Experiment 1 and a widely-used manipulation of self-construal priming.

### Materials and methods

#### Participants

Eighty-one healthy Chinese participants (42 females and 39 males; *M*_age_ = 21.7, *SD*_age_ = 2.7) participated in experiment 2. Participants were randomly assigned to an independent priming condition, an interdependent priming condition or a neutral priming condition. The participants had normal or corrected-to-normal visual acuity. None of them had a history of neurological or psychiatric disorders. The data from an additional eight participants were not analyzed because participants expressed suspicion about the threat manipulation at the end of the experiment.

#### Procedures and stimulus materials

Experiment 2 had a 3 (self-construal priming: independent priming, interdependent priming or neutral priming condition) × 2 (social-evaluative threat manipulation: threatening or nonthreatening feedback) mixed design, with repeated measures on the second factor.

Participants completed the procedures from Experiment 1 with some additional steps. Before the target experimental session (i.e., social-evaluative threat manipulation and self-evaluation), participants were first asked to complete a self-construal priming task (Sui and Han, 2007). In the independent priming condition, participants were asked to read two stories about countryside containing independent pronouns (e.g., *I, mine*) and to count the number of pronouns that appeared. Similarly, in the interdependent priming condition, participants were asked to read the same two stories with interdependent pronouns (e.g., *we, ours*) and count the number of pronouns that appeared. In addition, we also set a neutral priming condition where participants read two stories about the countryside that did not contain independent or interdependent pronouns. In this neutral condition, participants needed to count the number of certain nouns that appeared in the stories.

After completing the self-construal priming task, the participants received a standardized social-evaluative threat manipulation and completed a self-evaluation task used in Experiment 1. Participants received the threatening or nonthreatening feedback, which lasted 10 s. Across the experiment, there were 3 threatening and 3 nonthreatening pieces of feedback, which were randomly presented. After each feedback session, there was a block of 10 personality traits that needed to be evaluated. As in Experiment 1, at the end of the experiment, the Rosenberg Self-esteem Scale (RSES; Rosenberg, 1965), a 10-item instrument, was used to assess self-esteem. Moreover, participants were received the same debriefing interview procedure used in the previous study, which ensured that the participants really believed in our social-evaluative threat manipulation and had no suspicions about the experiment.

### Results

A one-way ANOVA, which was applied to the scores on the self-esteem scale data of the Experiment 2, showed no significant differences in the scores of the self-esteem scale among different self-construal priming conditions, *F*_(2, 78)_ = 1.624, *p* = 0.204, η_*p*_^2^ = 0.040 (see Table 1).

The magnitude of the above-average effect (the mean reverse-scored value of personality trait words evaluation, which indicates increased desirability) in the self-evaluation task was calculated for six experimental conditions and was submitted to a 3 (self-construal priming: independent priming, interdependent priming or neutral priming condition) × 2 (social-evaluative threat manipulation: threatening or nonthreatening feedback) mixed-design ANOVA, with the between-subjects factor of self-construal priming. The results revealed no main effects of self-construal priming or social-evaluative threat manipulation, *F*_(2, 78)_ = 0.063, *p* = 0.939, η_*p*_^2^ = 0.002; *F*_(1, 78)_ = 2.535, *p* = 0.115, η_*p*_^2^ = 0.031, respectively. However, we found a reliable self-construal priming × social-evaluative threat manipulation two-way interaction, *F*_(2, 78)_ = 11.384, *p* < 0.001, η_*p*_^2^ = 0.226 (see Table 1). A further simple effects analysis revealed a significant effect of social-evaluative threat manipulation in the independent self-construal priming, interdependent self-construal priming and neutral conditions, *F*_(1, 78)_ = 8.53, *p* = 0.005, η_*p*_^2^ = 0.109; *F*_(1, 78)_ = 11.65, *p* = 0.001, η_*p*_^2^ = 0.150; *F*_(1, 78)_ = 5.12, *p* = 0.026, η_*p*_^2^ = 0.064, respectively. We tested for replication of prior studies, which used Western participants, and showed that threatening feedback can cause an increase in the above-average effect (Beer et al., 2013; Hughes and Beer, 2013). The planned *t*-tests on simple effects showed that, for the independent self-construal priming condition, the magnitude of the above-average effect in the threatening feedback condition was significantly greater compared to those in the nonthreatening feedback condition, *t*_(26)_ = 3.23, *p* < 0.005, Cohen's *d* = 0.62. However, this replication only occurred in the independent self-construal priming condition. For the interdependent self-construal priming and neutral priming conditions, we failed to find that threatening feedback could cause an increase in the above-average effect compared to those caused by nonthreatening feedback. As opposed to the independent condition, after interdependent self-construal priming and neutral priming, the magnitude of the above-average effect in the nonthreatening feedback condition was significantly greater than those in the threatening feedback condition, *t*_(26)_ = −3.04, *p* = 0.005, Cohen's *d* = 0.59; *t*_(26)_ = −2.34, *p* < 0.05, Cohen's *d* = 0.45, respectively. These findings indicate that, following the independent self-construal priming, participants rated themselves as having significantly less undesirable personality traits when they were faced with the social-evaluative threat. However, this effect emphasizes that their desirability when faced with threatening feedback did not occur after participants received interdependent self-construal priming or neutral priming. Instead, participants rated themselves as having significantly less undesirable personality traits when they faced were with the nonthreatening feedback.

## Discussion

The current study aimed to observe whether East Asians have a different response pattern when faced with social threat, and then examine whether the different pattern was caused by different types of self-construal. To meet these objectives, we recruited Chinese participants to perform self-evaluation and self-construal priming tasks, with the above-average effect indicating the extent of the positive illusion of self during the self-evaluation. We compared the above-average effect of threatening and nonthreatening feedback conditions during a self-evaluation task and discovered that the Chinese participants rated themselves as having significantly greater above-average effect only when they were faced with the nonthreatening feedback (Experiment 1). Furthermore, we found an interaction between self-construal and the type of social-evaluative threat manipulation (Experiment 2). Following independent self-construal priming, the participants tended to deny their negative traits in an overemphasized way when faced with social-evaluative threats. Interestingly, this pattern of downplaying negative traits while under the influence of social threat did not appear after participants received interdependent self-construal priming or neutral priming. In contrast, participants even tended to evaluate themselves more desirably in the nonthreatening feedback condition. Taken together, these findings are consistent with our hypotheses that East Asians would engage in opposite patterns of self-evaluation when faced with a social threat, and people with different types of self-construal would have different patterns of self-evaluation when faced with social threats.

Consistent with previous above-average effect studies using Western participants, Chinese participants viewed themselves as having less negative personality traits compared to their peers (e.g., Yates et al., 1997; Acker and Duck, 2008; Beer and Hughes, 2010; Beer et al., 2010). However, the current study further found that, in comparison to the threatening feedback condition, Chinese participants tended to rate themselves as having significantly greater above-average effect when they were faced with the nonthreatening feedback condition. Different from previous studies, the current study found an opposite pattern (Vohs and Heatherton, 2004; vanDellen et al., 2011; Brown, 2012; Beer et al., 2013; Hughes and Beer, 2013), suggesting that the effect of social threat on self-evaluation is not culturally universal. Namely, Western participants tend to evaluate themselves more desirably in threatening situations. While, East Asians tend to exhibit this self-favoring bias only in nonthreatening situations but not in threatening situations. Our results converge with results from studies that examine the influence of social threats on self-related judgments. Just as previous studies have suggested that social threats may weaken the self-advantage effect of Chinese participants (Ma and Han, 2009; Liew et al., 2011), positive items may facilitate the self-advantage effect of Chinese participants (Ma and Han, 2010). Overall, the initial observations implicate that the potential cultural differences between East and West may exist in the processing of self-evaluation under social threat. More importantly, the current study further investigated the role of self-construal in self-evaluation under social-evaluative threat. Several studies suggest that culture can influence and form one's self-concept. In particular, Westerners tend to be more individualistic and have an independent self-construal. In contrast, East Asians tend to be more collectivistic and have an interdependent self-construal. Based on these views, in the second experiment, we tried to identify whether the cultural differences stem from the participants having different types of self-construal. In line with previous studies, we found a similar pattern when participants were primed by independent self-construal (Beer et al., 2013; Hughes and Beer, 2013). This finding was a conceptual replication of previous Western findings. Moreover, the most striking finding was that we found that the pattern of the above-average effect under the interdependent self-construal priming and neutral priming conditions contrasted with that under the independent self-construal priming condition. These results replicated the result of our first experiment, and further highlighted the effect of self-construal. The current findings have important implications for broadening investigations of self-evaluation under socially threatening environments. Most of the previous research on self-evaluation under socially threatening environment has primarily focused on identifying the behavioral performance and neural activation of the Western sample. These studies have concluded that people tend to emphasize their desirability when experiencing social threats, which can be regarded as a self-protection strategy. The current study further investigated another typical type of sample (i.e., East Asians) and suggests that whether a participant emphasizes his or her desirability in the face of social threats largely depends on how the participant views himself or herself (i.e., self-construal). Namely, the previous conclusion may apply merely to the participants who hold an independent self-construal. If the participants hold an interdependent self-construal, they would tend to emphasize their desirability in nonthreatening environments rather than threatening environments.

One possible argument is that, the reason that the results showed there were differences between the two groups is because the level of self-esteem of East Asians is lower than that of Westerners (Schmitt and Allik, 2005; Chiu and Hong, 2006). However, the current experiments have found that the Chinese participants report a similar self-esteem score and global magnitude of above-average effect as previous Western findings (e.g., Beer et al., 2013; Hughes and Beer, 2013). Namely, the Chinese participants may feel as positively toward themselves as Westerners do and have a similar level of self-esteem (Cai et al., 2007; Boucher et al., 2009). Based on these results, it seems that the experimental effect of the current study was not caused by the different levels of self-esteem.

Several previous studies have also found that people tend to emphasize the self's desirability in response to social-evaluative threats and that only individuals with low self-esteem or depression often serve as exceptions (Vohs and Heatherton, 2004; vanDellen et al., 2011), but our current results provided another exception. We recruited Chinese college students with normal self-esteem and that none of them had a history of neurological or psychiatric disorders. Thus, it is unlikely that individual difference factors would affect the results of the current study. If the different tendency of emphasizing the self's desirability in the face of social threats after different self-construal priming was not due to the participants in different conditions having different levels of self-esteem or depressive symptoms, why can self-construal priming modulate the pattern of self-evaluation under social threat? Across the current two experiments, we speculated that choosing different strategies for protecting the self with different types of self-construal might contribute to this difference. Humans are social animals. To maximize the likelihood and quality of survival in the social environment, an individual may tend to choose a self-protection strategy that is catered to their self-construal and cultural environment. As mentioned before, for people with an independent self-construal, the self is an autonomous entity, separate from other individuals (Markus and Kitayama, 1991; Heine, 2001), so they do not have to ruminate about other people's negative opinions or even fight the social-evaluative threat through emphasizing their desirability. However, for people with an interdependent self-construal, the self is a socially embedded entity with strong interconnectedness with others, and they often care very much about others' attitudes toward oneself. Therefore, to deny others' negative evaluations by emphasizing their desirability by leveraging threats is not a wise self-protection strategy.

Although, this study provides an initial step toward understanding self-evaluation and social threat in a cultural perspective, several limitations of the study should be noted. First, because only negative traits were selected as evaluative materials, our study provided a preliminary test of the modulating effect of self-construal priming on self-evaluation in the context of social threat. It is unclear whether the modulation found in the present study also exists with regard to positive traits. However, Beer et al. (2013) showed that, when participants evaluated themselves in threatening and nonthreatening condition, there were similar patterns between negative traits and common positive events. Future studies should systematically examine the modulating effect found in the present study by using positive materials. Second, in the current study we recruited Chinese participants to observe whether they have different response patterns when faced with social-evaluative threat. Although, we used the same paradigm as previous Western studies and observed the opposite pattern to previous findings, the current study did not recruit Western participants and compare their magnitude of above-average effect with Chinese participants within one experiment. Future studies should use revised paradigms, recruit the samples from two cultural environments and compare them directly. Finally, it has been found that self-evaluations made in response to social-evaluative threat increased activation in some brain regions such as OFC, mPFC, and amygdala (e.g., Flagan and Beer, 2013; Hughes and Beer, 2013). However, according the current study findings, it is unclear whether the cultural environment or self-construal can modulate the activation of specific brain regions. Based on the existing research findings, next step work may combine functional neuroimaging techniques and behavioral tasks to examine the possibility mentioned above.

In summary, the current study demonstrated the effects of cultural factor on self-evaluation under social threat. On one hand, by collecting Chinese samples, we found that compared to the Western studies, Chinese participants showed an opposite pattern to self-evaluation under social threat. That is, the participants only evaluated them themselves more positively when they faced with nonthreatening feedback. On the other hand, by manipulating self-construal and the type of social-evaluative threat in self-evaluation processing, we found that participants evaluated themselves in an especially flattering way to face the social-evaluative threat but only when they had been primed under the independent self-construal view. However, the phenomenon of emphasizing their desire to face the social-evaluative threat disappeared upon priming the participants for interdependent self-construal view. Our findings generally indicate that whether the participants emphasize their desirability in response to social threat largely depends on which cultural environment they live in, and how they view themselves. These findings may provide initial empirical evidence toward extending cognitive models of self-evaluation to social threat contexts by considering cultural factors.

## Ethics statement

This study was carried out in accordance with the recommendations of experimental guidelines from Ethics Committee of the School of Psychological and Cognitive Sciences, Peking University with written informed consent from all subjects. All subjects gave written informed consent in accordance with the Declaration of Helsinki. The protocol was approved by the Ethics Committee of the School of Psychological and Cognitive Sciences, Peking University.

## Author contributions

YW and TZ proposed the main research idea. TZ and SX made the research design and conducted the experiments. TZ designed the experimental materials and ran the statistics. TZ, YW, and SX made the discussion and wrote the manuscript. YJ took the charge of language revision. All authors reviewed the manuscript.

### Conflict of interest statement

The authors declare that the research was conducted in the absence of any commercial or financial relationships that could be construed as a potential conflict of interest. The reviewer, YZ, declared a shared affiliation, though no other collaboration, with the authors to the handling Editor.
